# Food Insecurity and Mental Health: A Moderated Mediation Analysis

**DOI:** 10.1111/cars.70009

**Published:** 2025-05-08

**Authors:** Lei Chai

**Affiliations:** ^1^ Department of Social Work and Social Administration The University of Hong Kong Hong Kong China

**Keywords:** food insecurity, marriage, mental health, sleep problems

## Abstract

Extensive research has demonstrated the negative impact of food insecurity on mental health; however, the mediating and moderating mechanisms underlying this relationship remain underexplored. Using data from the 2022 National Health Interview Survey (*N* = 25,703), this study investigates whether sleep problems mediate the relationship between food insecurity and mental health outcomes—specifically depressive and anxiety symptoms—and whether marital status moderates this relationship. The findings indicate that sleep problems partially mediate the effects of food insecurity on depressive and anxiety symptoms. In addition, the impact of sleep problems on these mental health outcomes is less severe among married individuals compared to their unmarried counterparts. However, marital status does not moderate the relationship between food insecurity and sleep problems, nor the relationship between food insecurity and mental health outcomes. The analysis of conditional indirect effects reveals a more pronounced mediation effect of sleep problems among unmarried individuals. These results suggest a partial protective role of marriage in mental health and underscore the importance of addressing sleep problems, particularly among unmarried individuals, in understanding the interplay between food insecurity, sleep problems, and mental health.

## FRENCH ABSTRACT

De nombreuses recherches ont démontré l'impact négatif de l'insécurité alimentaire sur la santé mentale. Cependant, les mécanismes médiateurs et modérateurs qui sous‐tendent cette relation restent sous‐explorés. En utilisant les données de la “National Health Interview Survey” de 2022 (N = 25 703), cette étude cherche à déterminer si les problèmes de sommeil influencent la relation entre l'insécurité alimentaire et les résultats en matière de santé mentale—en particulier les symptômes dépressifs et anxieux—et si l'état matrimonial modère cette relation. Les résultats indiquent que les problèmes de sommeil influencent partiellement les effets de l'insécurité alimentaire sur les symptômes de dépression et d'anxiété. En outre, l'impact des problèmes de sommeil sur ces effets sur la santé mentale est moins important chez les personnes mariées que chez les célibataires. Cependant, l'état matrimonial ne modère pas la relation entre l'insécurité alimentaire et les problèmes de sommeil, ni la relation entre l'insécurité alimentaire et les résultats en matière de santé mentale. L'analyse des effets indirects conditionnels révèle un effet de médiation plus prononcé des problèmes de sommeil chez les personnes non mariées. Ces résultats suggèrent un rôle protecteur partiel du mariage sur la santé mentale et soulignent l'importance d'aborder les problèmes de sommeil, en particulier chez les personnes non mariées, pour comprendre l'interaction entre l'insécurité alimentaire, les problèmes de sommeil et la santé mentale.

## 1 Introduction

Food insecurity remains a pressing concern in the United States. As of 2019, an estimated 10.5% of US households—approximately 13.7 million—experienced food insecurity (Coleman‐Jensen et al. 2021). A strong association between food insecurity and adverse mental health outcomes is well‐documented in the literature (Chai 2023a; Ciciurkaite and Brown 2018, 2022; Graham and Ciciurkaite 2023). However, the mechanisms linking food insecurity to mental health remain an area of active investigation. Drawing on the stress process model, and more specifically the stress proliferation perspective (Pearlin and Bierman 2013), this study theorizes how food insecurity, as a chronic stressor, can generate additional secondary stressors that contribute to poor mental health. We propose that sleep problems may serve as a key mediating factor in this relationship.

Economically‐related stressors are well‐established contributors to poor sleep (Bierman 2021; Chai and Lu 2025). In this vein, food insecurity—a salient dimension of economic hardship (Ciciurkaite and Brown 2022; Graham and Ciciurkaite 2023)—is increasingly linked to disrupted sleep patterns (Arenas et al. 2019; Mazloomi et al. 2023). At the same time, substantial evidence connects sleep problems to adverse mental health outcomes (Chai 2023b; Chai and Lu 2025; Frazier 2023; Scott et al. 2021). Despite these documented links, few nationally representative studies have explicitly tested whether sleep problems mediate the relationship between food insecurity and mental health. Investigating sleep in this context offers insight into how food insecurity—a persistent and psychologically taxing form of economically‐related strain—may influence mental health through stress‐related pathways.

**FIGURE 1:**
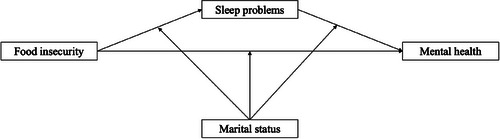
Conceptual model.

In addition to exploring the mediating role of sleep, this study examines marital status as a potential moderator of these dynamics. The stress process model emphasizes that social statuses such as marital status can condition the effects of stressors (Bierman 2009, 2012, 2014; Pearlin and Bierman 2013). Spouses often share and help mitigate stressful experiences, including those related to food insecurity or sleep problems, making marital support an important factor in buffering the effects of these stressors on mental health (Donnelly et al. 2019). However, prior research offers mixed findings on the role of marital status in this context (Chai 2023a; Ciciurkaite and Brown 2018), highlighting the need for further empirical inquiry. Using data from the 2022 National Health Interview Survey, this study tests whether sleep problems mediate the relationship between food insecurity and mental health—specifically, depressive and anxiety symptoms—and whether marital status moderates these associations. Figure 1 presents the conceptual framework guiding this investigation.

## 2 Background

### 2.1 Food Insecurity and Mental Health

Food insecurity involves not only the experience of physical hunger but also limited dietary variety and persistent concerns about food availability (Ciciurkaite and Brown 2022; Graham and Ciciurkaite 2023). Importantly, not all individuals facing economic challenges experience food insecurity in the same way. For instance, research shows that around one‐third of households living below the poverty line report no food insecurity (Wight et al. 2014). This discrepancy underscores the need to distinguish food insecurity from other economic indicators—such as poverty, income, and assets—when examining its effects on mental health.

A substantial body of research has established a strong association between food insecurity and a range of mental health outcomes (Chai 2023a; Ciciurkaite and Brown 2018, 2022; Graham and Ciciurkaite 2023). The stress process model offers a valuable framework for understanding these links, emphasizing how chronic and acute stressors, in conjunction with psychosocial resources, influence mental health outcomes (Pearlin and Bierman 2013). Within this framework, food insecurity is conceptualized as a chronic stressor—defined as the “limited or uncertain availability of nutritionally adequate and safe foods or limited or uncertain ability to acquire acceptable foods in socially acceptable ways” (Bickel et al. 2000, 6)—that contributes to mental health disparities.

Although food insecurity may be episodic for some, it is frequently understood in the sociological literature as a persistent and cumulative form of economic hardship (Ciciurkaite and Brown 2022; Graham and Ciciurkaite 2023). Chronic stressors, unlike acute life events, are defined by their ongoing nature and their potential to erode psychological well‐being over time (Pearlin and Bierman 2013). Economic hardship has long been recognized within the stress process model as a central example of chronic strain (Wheaton and Montazer 2010). Building on this framework, recent studies have identified food insecurity not only as a core dimension of economic hardship but also as a distinct, multifaceted source of chronic stress (Ciciurkaite and Brown 2022; Graham and Ciciurkaite 2023). Rather than reflecting temporary disruptions in food access, food insecurity can be conceptualized as a prolonged condition marked by persistent uncertainty, constrained choices, and reduced control over a basic human need. These features align closely with the sociological definitions of chronic strain (Pearlin and Bierman 2013) and reinforce the understanding of food insecurity as a pervasive, health‐eroding form of social stress (Ciciurkaite and Brown 2022; Graham and Ciciurkaite 2023). By situating food insecurity within the broader literature on economic hardship and chronic stress (Pearlin and Bierman 2013; Wheaton and Montazer 2010), this study advances sociological understanding of how an enduring and psychologically complex form of deprivation may shape mental health outcomes.

Evidence supporting the association between food insecurity and mental health spans a range of methodologies, including cross‐sectional studies (Chai 2023a), longitudinal research (Shepherd 2022), and meta‐analyses (Smith et al. 2023). These findings consistently demonstrate a robust link between food insecurity and mental health across diverse groups, including children (Thomas et al. 2019), adolescents (Poole‐Di Salvo et al. 2016), young adults (Graham and Ciciurkaite 2023), adults (Shepherd 2022), older adults (L. Chai and X. Chai 2025), people with disabilities (Brucker 2017), and marginalized racial/ethnic (Allen et al. 2018; Chai 2024) and sexual identity (Masa et al. 2024) groups. Collectively, this body of work underscores the widespread and far‐reaching impact of food insecurity on mental health.

### 2.2 The Mediating Role of Sleep Problems

While the relationship between food insecurity and mental health is well‐established, less attention has been paid to the mechanisms through which food insecurity shapes mental health outcomes. Building on the stress process model, and particularly the stress proliferation perspective, we theorize that food insecurity—a primary stressor—can lead to secondary stressors that further undermine well‐being (Pearlin and Bierman 2013). Recent studies have identified potential mediators in this process. For instance, Ciciurkaite and Brown (2022) found that social support and mastery partially explained the psychological effects of food insecurity. Similarly, Graham and Ciciurkaite (2023) revealed that social isolation and perceived life stress partially mediated the association between food insecurity and mental health among young American adults, while L. Chai and X. Chai (2025) identified community belonging as a mediator among older Canadian adults.

Expanding this line of research and drawing on sociological understandings of stress (Pearlin and Bierman 2013), we propose that sleep problems represent a socially patterned secondary stressor that mediates the relationship between food insecurity and mental health. Although sleep has often been studied through biomedical or psychological frameworks (e.g., Scott et al. 2021), an emerging body of research emphasizes that sleep is shaped by structural conditions and social inequalities rather than being solely an individual phenomenon. Research has linked sleep disturbances to factors such as income inequality, employment precarity, and housing instability (Burgard and Ailshire 2009; Johnson et al. 2018; Patel et al. 2024). Moreover, sleep is socially patterned by race, class, and gender (Burgard and Ailshire 2013; Grandner et al. 2016). Consistent with the stress process model, sleep problems can therefore be understood as socially patterned secondary stressors—embodied consequences of structural disadvantage that arise from individuals’ unequal exposure to chronic hardship. In this light, sleep problems are not simply personal health issues but reflect broader social patterns of deprivation and vulnerability (Hale et al. 2020), supporting their relevance as a key mechanism linking food insecurity to mental health outcomes.

Empirical studies support the role of food insecurity as a disruptor of sleep (Arenas et al. 2019; Mazloomi et al. 2023), suggesting that it undermines sleep quality through heightened uncertainty and the destabilization of daily routines (Bierman 2021; Bierman et al. 2018a). Although longitudinal evidence is limited, preliminary research among adolescents suggests a potential causal relationship (Lee et al. 2021). Research also consistently shows that sleep disturbances are strong predictors of mental health outcomes. Although much of this literature is cross‐sectional (Chai 2023b; Frazier 2023), several longitudinal studies and meta‐analyses provide compelling evidence that sleep problems precede and contribute to mental health conditions (Chai and Lu 2025; Scott et al. 2021). This relationship has been documented in various populations, including working adults (Foo and Doan 2023) and university students (Freeman et al. 2017). Although some studies suggest a bidirectional relationship (Rosenström et al. 2012; Saunders et al. 2023), growing theoretical and empirical work supports the causal role of sleep disruption, through mechanisms such as dysregulation of circadian rhythms, in predicting mental health outcomes (Frazier 2023).

Despite mounting evidence that sleep is consequential for mental health, no nationally representative studies have tested its role as a mediator in the association between food insecurity and mental health. Research on other chronic stressors offers insight. For instance, sleep problems mediate the impacts of everyday discrimination (Hisler and Brenner 2019) and financial strain (Chai and Lu 2025) on mental health in longitudinal studies, and cross‐sectional work finds that sleep problems help to explain the relationship between financial strain and depressive symptoms (Chai 2023b). While findings are mixed—Frazier (2023), for example, reported limited support for sleep mediation between shift work and depressive symptoms—the stress proliferation perspective nevertheless positions sleep disturbances as a plausible pathway through which stressors, including food insecurity, erode mental health (Pearlin and Bierman 2013). In doing so, we position sleep as a theoretically meaningful construct within the sociology of mental health—rather than as simply an individual‐level health outcome.

### 2.3 The Moderating Role of Marital Status

This study also explores whether marital status moderates the indirect effect of food insecurity on mental health via sleep problems. Marital status is a key social status within the stress process framework and has been shown to influence how individuals experience and respond to stressors (Bierman 2009, 2012, 2014; Pearlin and Bierman 2013). Several mechanisms underlie the protective role of marriage. Financially, marriage may confer greater stability and reduce exposure to stress through the pooling of resources and increased capacity to weather chronic strain (Chen et al. 2015). It can also increase access to health‐promoting resources, such as insurance and mental health services (Walker et al. 2015). Emotionally, marriage offers a critical source of support: spouses often share the emotional burden of chronic stressors, such as food insecurity and sleep difficulties, and can lessen their psychological toll(Chen et al. 2015; Reczek et al. 2020). Ross (1991, 832) describes this as providing “confidence, security, and self‐assurance” during times of challenge. Spouses may also help reduce mental health stigma and promote care‐seeking (Thoits and Link 2016; Reczek et al. 2020). In addition, marriage may enhance social integration by expanding one's access to a broader network of supportive ties (Still 2021). Finally, from the perspective of social control theory (Ciciurkaite and Brown 2022; Umberson 1992), marriage can promote healthier behaviors through mutual monitoring and regulation of stress‐related coping practices (Donnelly et al. 2019).

At the same time, food insecurity can place strain on social relationships. Individuals experiencing food insecurity often withdraw from social engagement due to stigma, shame, or the inability to participate in food‐centered gatherings (Meijs et al. 2020; Pineau et al. 2021; Purdam et al. 2016). In such situations, spouses may become the primary source of support, amplifying the protective effect of marriage. Empirical evidence consistently finds lower rates of mental health problems among married individuals (e.g., Bierman 2012). Studies have also found that marriage moderates the impact of other social stressors. For instance, marriage has been shown to weaken the association between functional limitations and depression among older men (Bierman 2012), and between neighborhood disorder and depressive symptoms (Bierman 2009). Similarly, Chai (2023b) found that marriage buffered the mental health effects of sleep problems in middle‐aged and older American adults.

Despite these insights, research on whether marital status moderates the effect of food insecurity remains limited and inconclusive. Ciciurkaite and Brown (2018), using data from the National Health and Nutrition Examination Survey (NHANES), found little evidence of moderation—possibly due to the inclusion of cohabiting individuals in the “married” group. In contrast, Chai (2023a), utilizing data from the 2017 to 2018 Canadian Community Health Survey (CCHS), found that married individuals reported a weaker association between food insecurity and poorer self‐rated mental health. These mixed findings highlight the importance of further investigation into how marital status intersects with structural disadvantage to shape mental health outcomes.

### 2.4 The Current Study

Informed by the theoretical foundations of the stress process model and relevant empirical evidence, this study tests two hypotheses using a recent nationally representative sample of American adults. *First, we expect that sleep problems will at least partially mediate the association between food insecurity and mental health problems* (**Hypothesis 1**). *Second, we expect that marital status will moderate the direct effects of food insecurity and its indirect effects through sleep problems on mental health, so that the negative effects of food insecurity and sleep problems are expected to be less pronounced among married individuals* (**Hypothesis 2**).

## 3 Methods

### 3.1 Data

This study used data from the 2022 National Health Interview Survey (NHIS), conducted by the US Census Bureau and the National Center for Health Statistics (NCHS). The NHIS is an annual survey designed to collect comprehensive information on the health and well‐being of the American population using a multistage probability sampling design. Extensive details regarding the study design and sampling frame are available in documentation provided by the NCHS (2023). The 2022 data file included responses from 27,651 adults, with a response rate of 47.7%. This study involved secondary analyses of publicly available, de‐identified data, exempting it from ethical review board approval. Analyses were restricted to adults aged 18 years and older. Missing values were minimal, ranging from sex (0.01%) to food insecurity (4.58%), and were handled using listwise deletion. The final analytic sample size consisted of 25,703 adults.

### 3.2 Measures

Depressive symptoms were measured using the Patient Health Questionnaire‐8 (PHQ‐8). Respondents indicated how often they were bothered by the following statements in the past 2 weeks: “little interest or pleasure in doing things,” “feeling down, depressed, irritable, or hopeless,” “trouble falling or staying asleep, or sleeping too much,” “feeling tired or having little energy,” “poor appetite or overeating,” “feeling bad about yourself, or that you are a failure, or have let yourself or your family down,” “trouble concentrating on things, such as reading the newspaper or watching television,” and “moving or speaking so slowly that other people could have noticed? Or the opposite, being so fidgety or restless that you have been moving around a lot more than usual.” Responses were recoded as “not at all” (0), “several days” (1), “more than half the days” (2), and “nearly every day” (3). To reduce multicollinearity with the measure of sleep problems, the sleep‐related item was excluded from the analysis (Frazier 2023). Scores were summed across the remaining seven items, yielding a range from 0 to 21 (Cronbach's alpha = 0.83).

Anxiety symptoms were assessed using the Generalized Anxiety Disorder 7‐item (GAD‐7) scale. Respondents reported how often they were bothered by the following statements in the past 2 weeks: “feeling nervous, anxious, or on edge,” “not being able to stop or control worrying,” “worrying too much about different things,” “trouble relaxing,” “being so restless that it is hard to sit still,” “becoming easily annoyed,” and “feeling afraid as if something awful might happen.” Responses were recoded as “not at all” (0), “several days” (1), “more than half the days” (2), and “nearly every day” (3). Scores were summed, resulting in a range from 0 to 21 (Cronbach's alpha = 0.89).

Sleep problems were measured using four items assessing experiences in the past 30 days (Chai 2023b): “wake up well rested,” “trouble falling asleep,” “trouble staying asleep,” and “take medication for sleep.” Responses were recoded as “never” (1), “some days” (2), “most days” (3), and “every day” (4). The first item was reverse‐coded, and scores were summed to create an index, with higher scores indicating greater sleep problems (Cronbach's alpha = 0.64).

Food insecurity was measured using the 10‐item U.S. Adult Food Security Survey Module (Masa et al. 2024), which assesses the experiences of “worry food would run out,” “food did not last,” “could not afford to eat balanced meals,” “cut the size of meals or skip meals,” “how many days did you/adults in the family cut the size of meals or skip meals,” “eat less than should,” “ever hungry because not enough money for food,” “lose weight because not enough money for food,” “not eat for a whole day,” and “how many days not eat.”

The complex scoring process for this measure follows the Economic Research Service (ERS)—U.S. Department of Agriculture guidelines (2012). Interested readers can refer to ERS (2012) for full documentation. In line with prior research, we used the preconstructed NHIS variable and recoded it into a binary variable (Ciciurkaite and Brown 2022; Chai 2023a), with 1 = “low/very low food secure” and 0 = “food secure.” This approach enhances statistical power and ensures greater reliability when assessing the moderating role of marital status.

Legal marital status was originally coded as “separated,” “divorced,” “married,” “single/never married,” or “widowed.” These categories were recoded into a binary variable distinguishing between “married” and “unmarried” (Bierman 2012, 2014; Chai 2023a).

Sociodemographic characteristics associated with food insecurity and mental health were controlled for in the analysis. Sex was coded as “male” and “female.” Age was measured in years. Race/ethnicity was recoded as “Hispanic,” “Non‐Hispanic White only,” “Non‐Hispanic Black/African American only,” “Non‐Hispanic Asian only,” and “Other.” Education was recoded as “less than high school,” “high school,” “some college,” “bachelor,” and “above bachelor.” Employment status was coded as “yes” and “no.” Income‐to‐poverty ratio, used as a control for economic status (Ciciurkaite and Brown 2018), was recoded as “less than 1,” “1 to 1.99,” “2 to 3.99,” and “4 and above” (Williams and Do 2021). This variable is calculated as the ratio of a family's income to the poverty threshold defined by the US Census Bureau. The region was categorized as “Northeast,” “Midwest,” “South,” and “West.”

Hours of sleep, measured as a continuous variable, were also included as a control. While short sleep duration—typically defined as less than 6 h—is often treated as a dimension of sleep problems (Chai and Lu 2025), its binary measurement differs from the ordinal scales used for the other dimensions. This difference made it unfeasible to integrate short sleep duration into the same index. Instead, hours of sleep were included as a control variable to account for its unique contribution to the outcome while maintaining the integrity of the sleep problems index. Table 1 presents the descriptive statistics of the variables included in the study.

**TABLE 1:** Descriptive statistics of selected variables in the analysis (*n* = 25,703).

	Mean/%	S.D.	Range
Depressive symptoms	2.15	4.12	0–21
Anxiety symptoms	2.31	4.79	0–21
Sleep problems	3.17	2.86	0–12
Food insecurity			
Yes	7.83		
No (ref)	92.17		
Married			
Yes	52.16		
No (ref)	47.84		
Sex			
Male	48.68		
Female (ref)	51.32		
Age	48.12	22.00	18–85
Race/ethnicity			
Hispanic	17.00		
White (ref)	62.92		
Black	11.22		
Asian	6.07		
Other	2.79		
Education			
Less than high school (ref)	12.76		
High school	24.64		
Some college	29.62		
Bachelor	20.41		
Above bachelor	12.57		
Employment			
Yes	63.76		
No (ref)	36.24		
Income to poverty ratio			
Less than 1 (ref)	9.39		
1 to 1.99	17.58		
2 to 3.99	28.99		
4 or above	44.04		
Region			
Northeast (ref)	17.26		
Midwest	20.88		
South	38.04		
West	23.81		
Hours of sleep	7.12	1.64	1–24

*Note*: Means or percentages are weighted, but *n* is unweighted.

### 3.3 Analytical Strategy

Data analysis was conducted using the PROCESS macro developed by Hayes (2013). This tool was employed to test the proposed hypotheses, following precedent in similar research (e.g., Cava et al. 2023; Chai 2023b). Specifically, the analysis focused on simple mediation (Model 4) and moderated mediation (Model 59). PROCESS is widely used for estimating mediation and moderated mediation effects due to its flexibility and reliance on bootstrapping methods, which generate robust confidence intervals for indirect effects. This approach addresses key limitations of earlier methods, such as the Baron and Kenny (1986) framework, by emphasizing the significance of indirect effects based on bootstrapped confidence intervals. While alternative methods—such as structural equation modeling (SEM) or the Karlson–Holm–Breen (KHB) method—may be suitable for mediation analysis alone, PROCESS offers an integrated and efficient solution for simultaneously examining mediation and moderated mediation effects within a single framework.

The methodology used in this study accommodates categorical variables, including binary ones, as both independent variables and moderators. To enhance the accuracy of confidence intervals for the indirect effects and maintain the balance between Type I and Type II errors, a bootstrapping method was employed (Mallinckrodt et al. 2006). A total of 5000 bootstrapped samples were drawn for the analysis. Mediation and moderated mediation effects were considered significant if the 95% bias‐corrected confidence intervals did not include zero. All statistical analyses were conducted using SPSS software, version 29.

## 4 Results

The analysis results, presented in Table 2, shed light on the relationships between food insecurity, sleep problems, and mental health, adjusting for a full set of control variables. Model 1 revealed that food insecurity was significantly associated with higher levels of depressive symptoms (*b* = 2.576, *p* < 0.001). Similarly, Model 2 produced a significant association between food insecurity and higher levels of anxiety symptoms (*b* = 2.759, *p* < 0.001). Model 3 showed that food insecurity was also associated with greater sleep problems (*b* = 1.195, *p* < 0.001). Models 4 and 5 demonstrated significant associations between sleep problems and higher levels of depressive symptoms (*b* = 0.659, *p* < 0.001) and anxiety symptoms (*b* = 0.702, *p* < 0.001), respectively. The associations between food insecurity and both depressive symptoms (*b* = 1.789, *p* < 0.001) and anxiety symptoms (*b* = 1.920, *p* < 0.001) remained significant even after accounting for sleep problems. Notably, the effect of food insecurity on depressive symptoms decreased from 2.576 in Model 1 to 1.789 in Model 4. Similarly, the effect of food insecurity on anxiety symptoms decreased from 2.759 in Model 2 to 1.920 in Model 5.

**TABLE 2:** Mediation analysis (*n* = 25,703).

	Model 1:		Model 2:		Model 3:		Model 4:		Model 5:	
	Depressive symptoms		Anxiety symptoms		Sleep problems		Depressive symptoms		Anxiety symptoms	
	*b*	95% CI	*b*	95% CI	*b*	95% CI	*b*	95% CI	*b*	95% CI
Food insecurity (= 1)	2.576^***^	(2.422, 2.731)	2.759^***^	(2.586, 2.933)	1.195^***^	(1.084, 1.307)	1.789^***^	(1.652, 1.927)	1.920^***^	(1.763, 2.076)
Sleep problems							0.659^***^	(0.644, 0.673)	0.702^***^	(0.685, 0.719)
Intercept	6.544		8.600		8.240		1.118		2.813	
$R^{2}$	0.118		0.126		0.162		0.317		0.303	

*Note*: All models include the following control variables: marital status, sex, age, race/ethnicity, education, employment status, income‐to‐poverty ratio, region, and sleep hours.

*** *p* < 0.001.

Bootstrapped indirect effect analyses confirmed significant indirect relationships between food insecurity and both mental health outcomes through sleep problems. For depressive symptoms, the indirect effect was significant (ab = 0.787, 95% CI = 0.698, 0.875). For anxiety symptoms, the indirect effect was also significant (ab = 0.840, 95% CI = 0.745, 0.936). These results collectively supported Hypothesis 1, suggesting that food insecurity is linked to increased sleep problems, which subsequently contribute to higher levels of depressive and anxiety symptoms.

**FIGURE 2:**
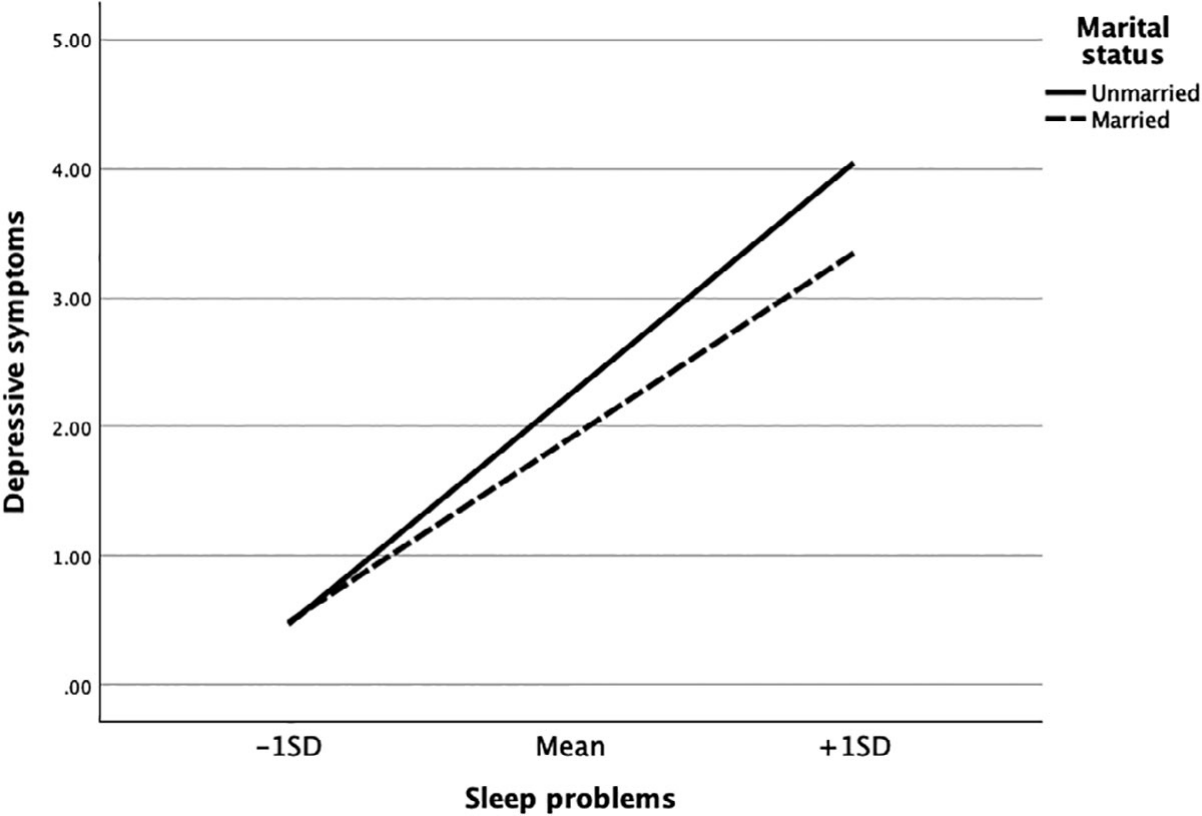
The moderating effects of marital status in the association between sleep problems and depressive symptoms.

**FIGURE 3:**
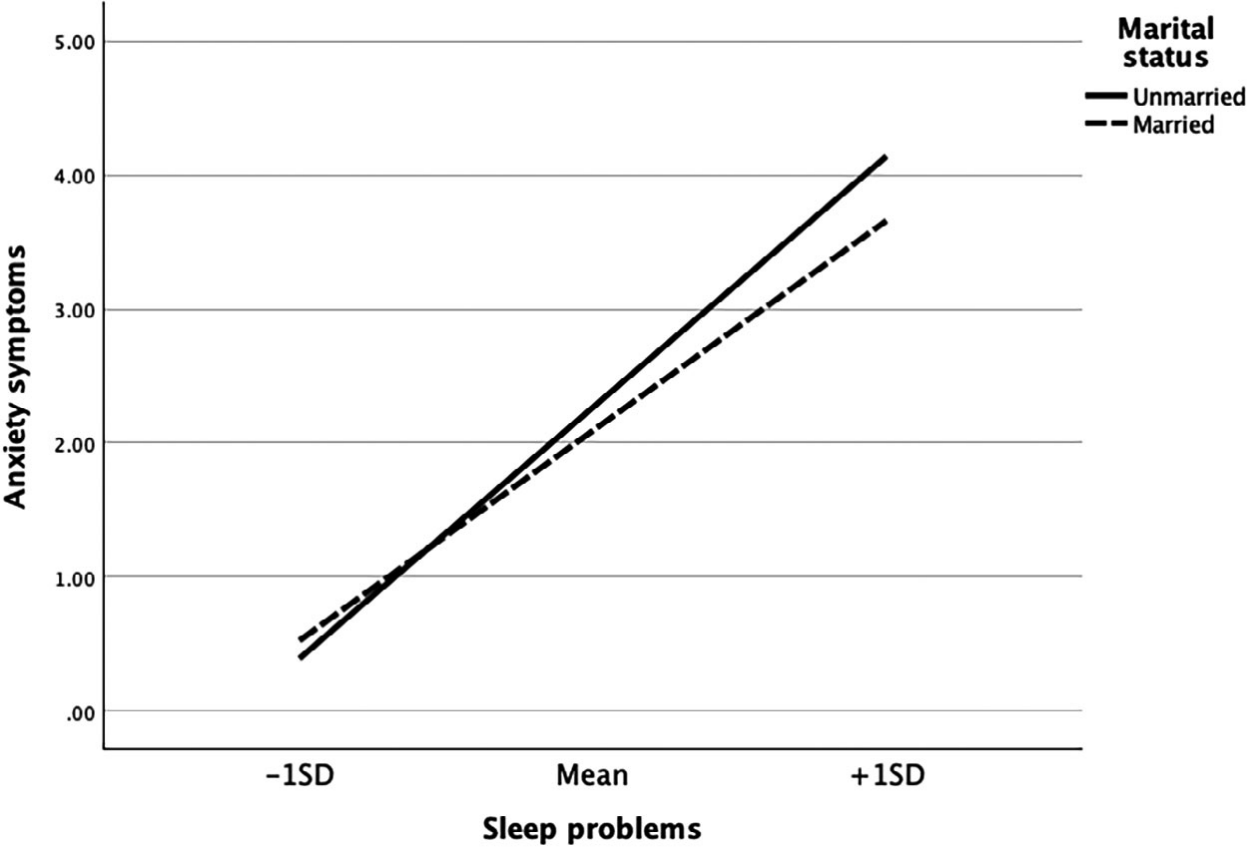
The moderating effects of marital status in the association between sleep problems and anxiety symptoms.

Table 3 investigates the moderating role of marital status in the relationships between food insecurity, sleep problems, and mental health. The analysis found no evidence that marital status moderated the association between food insecurity and sleep problems (*b* = −0.107, *p* > 0.05). Similarly, marital status did not significantly moderate the associations between food insecurity and depressive symptoms (b = 0.032, *p* > 0.05) or anxiety symptoms (*b* = 0.036, *p* > 0.05). However, marital status significantly moderated the relationships between sleep problems and depressive symptoms (b = −0.145, *p* < 0.001) and between sleep problems and anxiety symptoms (*b* = −0.124, *p* < 0.001). These findings suggest that the positive effects of sleep problems on depressive and anxiety symptoms were weaker among married individuals compared to unmarried ones. Figures 2 and 3 illustrate these two significant interaction effects.

**TABLE 3:** Moderated mediation analysis (*n* = 25,703).

	*b*	95% CI
Mediator variable model (outcome: sleep problems)		
Food insecurity (= 1)	1.224^***^	(1.095, 1.353)
Married (= 1)	−0.161^***^	(−0.222, −0.101)
Food insecurity × married	−0.107	(−0.346, 0.132)
Intercept	8.237	
$R^{2}$	0.162	
Dependent variable model (outcome: depressive symptoms)		
Food insecurity (= 1)	1.745^***^	(1.585, 1.904)
Sleep problems	0.721^***^	(0.702, 0.740)
Married (= 1)	0.125^*^	(0.011, 0.239)
Food insecurity × married	0.032	(−0.262, 0.326)
Sleep problems × married	−0.145^***^	(−0.173, −0.117)
Intercept	0.885	
$R^{2}$	0.319	
Conditional indirect effect analysis		
Unmarried	0.883	(0.772, 0.998)
Married	0.644	(0.501, 0.796)
Dependent variable model (outcome: anxiety symptoms)		
Food insecurity (= 1)	1.879^***^	(1.698, 2.061)
Sleep problems	0.756^***^	(0.734, 0.778)
Married (= 1)	0.225^***^	(0.094, 0.355)
Food insecurity × married	0.036	(−0.298, 0.370)
Sleep problems × married	−0.124^***^	(−0.156, −0.092)
Intercept	2.613	
$R^{2}$	0.305	
Conditional indirect effect analysis		
Unmarried	0.925	(0.803, 1.045)
Married	0.706	(0.550, 0.873)

*Note*: All models include the following control variables: sex, age, race, education, employment status, income‐poverty ratio, region, and sleep hours.

*** *p* < 0.001;*p <0.05.

The bias‐corrected percentile bootstrap analysis revealed significant indirect relationships between food insecurity and depressive symptoms through sleep problems for both unmarried (*b* = 0.883, 95% CI = 0.772, 0.998) and married (*b* = 0.644, 95% CI = 0.501, 0.796) individuals. The difference in these conditional indirect effects was statistically significant, as indicated by the index of moderated mediation (*b* = −0.239, 95% CI = −0.414, −0.058). Similarly, the indirect relationship between food insecurity and anxiety symptoms through sleep problems was significant for both unmarried (*b* = 0.925, 95% CI = 0.803, 1.045) and married (*b* = 0.706, 95% CI = 0.550, 0.873) individuals. The difference in these conditional indirect effects was also significant, as indicated by the index of moderated mediation (*b* = −0.219, 95% CI = −0.408, −0.025). These findings collectively provide partial support for Hypothesis 2.

## 5 Discussion and Conclusion

By leveraging data from the 2022 National Health Interview Survey, this study advances our understanding of the relationship between food insecurity and mental health. It introduces novel aspects by examining the potential mediating role of sleep problems and the moderating role of marital status in a nationally representative sample of American adults. Consistent with previous research (Chai 2023a; Ciciurkaite and Brown 2018, 2022; Graham and Ciciurkaite 2023), the study confirms the detrimental effects of food insecurity on depressive and anxiety symptoms. Several important insights emerge from this analysis.

One key contribution of this study is the exploration of sleep problems as a mediator in the food insecurity—mental health relationship. Previous studies have focused on various mediating pathways (L. Chai and X. Chai 2025; Ciciurkaite and Brown 2022; Graham and Ciciurkaite 2023), but the specific role of sleep problems in this context has not been extensively investigated. Consistent with the stress proliferation perspective (Pearlin and Bierman 2013), this study identifies food insecurity as a primary stressor that indirectly influences higher depressive and anxiety symptoms through the secondary stressor of sleep problems. This finding aligns with theoretical perspectives linking chronic stress from food insecurity to sleep disruption through physiological responses (Bierman 2021; Bierman et al. 2018a). Although systematic reviews and meta‐analyses have hinted at this connection (Arenas et al. 2019; Mazloomi et al. 2023), empirical studies have often been limited to specific demographics, such as adolescents (Osei Bonsu et al. 2023), young adults (Nagata et al. 2019), older adults (Arzhang et al. 2024), low‐income households (Troxel et al. 2020), and racial/ethnic minorities (Jordan et al. 2016). Two studies on the general population have used datasets preceding 2019 (Alhasan et al. 2023; Jacob et al. 2023). This study contributes newer evidence by employing recent data from the 2022 National Health Interview Survey.

Furthermore, this study finds that sleep problems contribute to higher depressive and anxiety symptoms, even after controlling for food insecurity, suggesting that both sleep problems and food insecurity independently affect mental health outcomes. This finding raises the possibility that disrupted circadian rhythms from sleep problems can adversely impact mental health (Frazier 2023; Pandi‐Perumal et al. 2020). While this study draws on Frazier's (2023) work to incorporate the causal direction from sleep problems to mental health, this explanation—attributing effects to circadian rhythm disruptions—is not grounded in sociological theory. This highlights a broader limitation: the scarcity of sociological frameworks addressing the causal direction between sleep problems and mental health. Future research should fill in this gap in the literature by developing and applying sociological theories to better understand how sleep and mental health are interconnected within the context of stress proliferation.

While this study highlights the independent contributions of sleep problems and food insecurity to mental health, it also raises questions about potential reverse causality and the bidirectional nature of these relationships. For instance, a recent cross‐sectional study suggests that mental health indicators mediate the association between food insecurity and sleep problems (Jacob et al. 2023). However, reliance on cross‐sectional data limits definitive conclusions about causality. Substantial evidence points to a bidirectional relationship between sleep problems and mental health (Rosenström et al. 2012; Saunders et al. 2023). Investigating the causal sequence between the two remains a priority for future research.

Extensive research highlights the mental health benefits of marriage, either through direct effects (Simon 2002; Williams 2003) or as a stress‐moderating factor (Bierman 2012; Chai 2023a, 2023b). However, findings of marital status as a moderator in the context of food insecurity have been inconsistent. While one study identified a protective role of marriage (Chai 2023a), another found no such effects (Ciciurkaite and Brown 2018). Methodological differences may explain these discrepancies, particularly the inclusion of cohabiting individuals in some studies (Ciciurkaite and Brown 2018). Carr and Springer (2010) emphasize the importance of distinguishing between married and cohabiting individuals, as marriage offers unique health benefits compared to cohabitation.

Unexpectedly, this study finds that marital status does not significantly moderate the effects of food insecurity on sleep problems, depressive symptoms, or anxiety symptoms. This contradicts literature supporting the various benefits of marriage, including financial (Chen et al. 2015; Walker et al. 2015), emotional (Chen et al. 2015; Reczek et al. 2020), and social (Umberson 1992) aspects. One possible explanation lies in gender‐specific variations in how marital status affects individuals facing food insecurity. Research shows that marriage often benefits men more than women in terms of well‐being (Bierman 2012; Kiecolt‐Glaser and Newton 2001). Wives typically provide emotional support in marriage, whereas women are more likely to seek support outside the marital relationship (Donnelly et al. 2019). Recent research supports this, indicating that marriage weakens the adverse effects of food insecurity on self‐rated mental health, but this protective effect is evident among men and not women when considering a three‐way interaction with gender (Chai 2023a). However, conducting three‐way interactions within a moderated mediation framework is not standard practice, underscoring the need for future studies to more explicitly explore the moderating roles of marital status and gender.

Despite these complexities, this study does show a significant role for marriage in reducing the harmful effects of sleep problems on depressive and anxiety symptoms, consistent with existing research (Chai 2023b). It underscores the importance of analyzing marital status as a moderating factor in relation to primary and secondary stressors (Bierman 2012). Moving forward, research should also examine how marital relationship quality influences these outcomes (Barr et al. 2013). Rather than lessening stress, low‐quality marriages, characterized by reduced companionship and increased conflict (Chen et al. 2015), may become stressors themselves. Evidence links low‐quality marital relationships to more severe mental health issues (Still 2021), including anxiety and depression (Choi and Marks 2008). Future research should explore how marital relationship quality interacts with primary and secondary stressors, such as food insecurity and sleep problems, to influence mental health outcomes.

This study provides valuable insights but is not without limitations. The cross‐sectional design of the NHIS data inhibits the ability to establish causality in the examined relationships. Utilizing longitudinal data would be more effective for understanding the causal dynamics involved in the mediating role of sleep problems and the moderating role of marital status in the relationship between food insecurity and mental health. Another limitation stems from the use of self‐reported measures, which may introduce recall bias. Although objective measures for sleep problems, such as actigraphy, could reduce measurement bias, they are often impractical due to their time‐consuming nature and high costs, potentially resulting in nonrepresentative samples. Notably, most nationally representative surveys, including the NHIS, rely on self‐reported data for mental health and associated behaviors like sleep problems (Frazier 2023). In addition, the somewhat low Cronbach's alpha value (0.64) for the sleep problems index warrants attention, although it aligns with values reported in previous literature (Bierman et al. 2018b; Chai 2023b; Chen et al. 2015). Another limitation involves the specificity of the marital status measure. While this study distinguishes between married and unmarried individuals, it does not differentiate among nonmarital statuses, such as divorced, widowed, or never married individuals. Future research could enrich understanding by examining these distinct groups, shedding light on the complex relationships between various marital statuses, sleep problems, and mental health outcomes in the context of food insecurity.

Despite these limitations, this study makes significant contributions to the existing literature by illuminating the mediating role of sleep problems and the moderating role of marital status in the relationship between food insecurity and mental health. Specifically, it sheds light on the mediation by sleep problems in linking food insecurity to mental health outcomes. Furthermore, it reveals that the adverse impacts of sleep problems on mental health are mitigated among married individuals, suggesting a potential protective effect of marriage. Consequently, this study deepens understanding of the complex interplay between food insecurity, sleep, and mental health, while highlighting the critical influence of marital status within these dynamics.

The findings of this study underscore several policy implications, advocating for interventions aimed at alleviating food insecurity and sleep issues to improve mental health outcomes. Policymakers are encouraged to enhance access to food assistance programs and support initiatives that promote affordable and nutritious food choices, addressing both stressful events and chronic strains associated with food insecurity. Moreover, public health interventions should prioritize promoting healthy sleep habits through education and expanding access to professional support for sleep disorders, recognizing the significant mediating role of sleep problems. Insights into the protective role of marriage further suggest that fostering strong social support systems, including relationship education and counseling services, could bolster resilience against the mental impacts of food insecurity. Tailored support for unmarried individuals, who face greater vulnerability, should include expanded mental health services and social programs to enhance social connectivity.

## Ethics Statement

The present study uses a publicly available microdata file of the 2022 NHIS, which does not require ethics board approval.

## Conflicts of Interest

The author declares no conflicts of interest.

## References

[cars70009-bib-0001] Alhasan, D. M. , N. M. Riley , W. B. Jackson II , and C. L. Jackson . 2023. “Food Insecurity and Sleep Health by Race/Ethnicity in the United States.” Journal of Nutritional Science 12: e59. 37252683 10.1017/jns.2023.18 PMC10214135

[cars70009-bib-0002] Allen, N. L. , B. J. Becerra , and M. B. Becerra . 2018. “Associations Between Food Insecurity and the Severity of Psychological Distress Among African‐Americans.” Ethnicity & Health 23, no. 5: 511–520. 28140616 10.1080/13557858.2017.1280139

[cars70009-bib-0003] Arenas, D. J. , A. Thomas , J. Wang , and H. M. DeLisser . 2019. “A Systematic Review and Meta‐Analysis of Depression, Anxiety, and Sleep Disorders in US Adults With Food Insecurity.” Journal of General Internal Medicine 34: 2874–2882. 31385212 10.1007/s11606-019-05202-4 PMC6854208

[cars70009-bib-0004] Arzhang, P. , N. Sadeghi , F. A. Harchegani , et al. 2024. “Associations Between Food Insecurity and Sleep Duration, Quality, and Disturbance Among Older Adults From Six Low‐ and Middle‐income Countries.” Journal of Nutrition, Health and Aging 28, no. 1: 100018.10.1016/j.jnha.2023.10001838267148

[cars70009-bib-0005] Baron, R. M. , and D. A. Kenny . 1986. “The Moderator–Mediator Variable Distinction in Social Psychological Research: Conceptual, Strategic, and Statistical Considerations.” Journal of Personality and Social Psychology 51, no. 6: 1173–1182. 3806354 10.1037//0022-3514.51.6.1173

[cars70009-bib-0006] Barr, A. B. , E. Culatta , and R. L. Simons . 2013. “Romantic Relationships and Health Among African American Young Adults: Linking Patterns of Relationship Quality Over Time to Changes in Physical and Mental Health.” Journal of Health and Social Behavior 54, no. 3: 369–385. 23657713 10.1177/0022146513486652

[cars70009-bib-0007] Bickel, G. , M. Nord , C. Price , W. Hamilton , and J. Cook . 2000. “Guide to Measuring Household Food Security.” U.S. Department of Agriculture, Food and Nutrition Service.

[cars70009-bib-0008] Bierman, A. 2009. “Marital Status as Contingency for the Effects of Neighborhood Disorder on Older Adults' Mental Health.” Journals of Gerontology: Series B 64B, no. 3: 425–434. 10.1093/geronb/gbp010 PMC2905133 19251881

[cars70009-bib-0009] Bierman, A. 2012. “Functional Limitations and Psychological Distress: Marital Status as Moderator.” Society and Mental Health 2, no. 1: 35–52.

[cars70009-bib-0010] Bierman, A. 2014. “Is Marital Status a Critical Contingency in the Relationship Between Physical Limitations and Subjective Well‐Being Among Japanese Adults?” Journal of Family Issues 35, no. 14: 1876–1897.

[cars70009-bib-0011] Bierman, A. 2021. “Why Have Sleep Problems in Later‐Midlife Grown Following the Great Recession? A Comparative Cohort Analysis.” Journals of Gerontology: Series B 76, no. 5: 1005–1014. 10.1093/geronb/gbaa034 32227082

[cars70009-bib-0012] Bierman, A. , Y. Lee , and S. Schieman . 2018a. “Chronic Discrimination and Sleep Problems in Late Life: Religious Involvement as Buffer.” Research on Aging 40, no. 10: 933–955. 29580186 10.1177/0164027518766422

[cars70009-bib-0013] Bierman, A. , Y. Lee , and S. Schieman . 2018b. “Neighborhood Disorder and Sleep Problems in Older Adults: Subjective Social Power as Mediator and Moderator.” Gerontologist 58, no. 1: 170–180. 28472476 10.1093/geront/gnx049

[cars70009-bib-0014] Brucker, D. L. 2017. “The Association of Food Insecurity With Health Outcomes for Adults With Disabilities.” Disability and Health Journal 10, no. 2: 286–293. 28017283 10.1016/j.dhjo.2016.12.006

[cars70009-bib-0015] Burgard, S. A. , and J. A. Ailshire . 2009. “Putting Work to Bed: Stressful Experiences on the Job and Sleep Quality.” Journal of Health and Social Behavior 50, no. 4: 476–492. 20099452 10.1177/002214650905000407 PMC3320737

[cars70009-bib-0016] Burgard, S. A. , and J. A. Ailshire . 2013. “Gender and Time for Sleep Among US Adults.” American Sociological Review 78, no. 1: 51–69. 25237206 10.1177/0003122412472048 PMC4164903

[cars70009-bib-0017] Carr, D. , and K. W. Springer . 2010. “Advances in Families and Health Research in the 21st Century.” Journal of Marriage and Family 72, no. 3: 743–761.

[cars70009-bib-0018] Cava, M. J. , I. Castillo , S. Buelga , and I. Tomás . 2023. “Relationships Among Romantic Myths, Tolerant Attitudes Toward Abuse, and Teen Dating Violence Victimization: The Moderator Role of Gender.” Youth & Society 55, no. 8: 1542–1567.

[cars70009-bib-0019] Chai, L. 2023a. “Food Insecurity and Health: Marital Status and Gender Variations.” Family & Community Health 46, no. 4: 242–249. 37703512 10.1097/FCH.0000000000000377

[cars70009-bib-0020] Chai, L. 2023b. “Financial Strain and Psychological Distress Among Middle‐Aged and Older Adults: A Moderated Mediation Model.” Journal of Gerontological Social Work 66, no. 8: 1120–1132. 37139587 10.1080/01634372.2023.2207611

[cars70009-bib-0021] Chai, L. 2024. “Food Insecurity and Its Association With Multiple Health Outcomes Among Indigenous Peoples in Canada: The Buffering Role of Culture‐Based Resources.” Ethnicity & Health 29, no. 3: 371–394. 38297918 10.1080/13557858.2024.2311419

[cars70009-bib-0022] Chai, L. , and X. Chai . 2025. “Unpacking the Association Between Food Insecurity and Mental Health Disorders Among Older Adults.” Journal of Aging and Health 8982643251314066. 39902548 10.1177/08982643251314066

[cars70009-bib-0023] Chai, L. , and Z. Lu . 2025. “The Association Between Financial Strain and Mental Health: The Mediating and Moderating Roles of Sleep Problems in the UK Household Longitudinal Study (UKHLS).” Journal of Affective Disorders 377: 245–253. 39983776 10.1016/j.jad.2025.02.060

[cars70009-bib-0024] Chen, J. H. , L. J. Waite , and D. S. Lauderdale . 2015. “Marriage, Relationship Quality, and Sleep Among US Older Adults.” Journal of Health and Social Behavior 56, no. 3: 356–377. 26272988 10.1177/0022146515594631 PMC4677485

[cars70009-bib-0025] Choi, H. , and N. F. Marks . 2008. “Marital Conflict, Depressive Symptoms, and Functional Impairment.” Journal of Marriage and Family 70, no. 2: 377–390. 18698378 10.1111/j.1741-3737.2008.00488.x PMC2507765

[cars70009-bib-0026] Ciciurkaite, G. , and R. L. Brown . 2018. “Food Insecurity, Psychological Distress and Alcohol Use: Understanding the Salience of Family Roles for Gender Disparities.” Health Sociology Review 27, no. 3: 294–311.

[cars70009-bib-0027] Ciciurkaite, G. , and R. L. Brown . 2022. “The Link Between Food Insecurity and Psychological Distress: The Role of Stress Exposure and Coping Resources.” Journal of Community Psychology 50, no. 3: 1626–1639. 34735724 10.1002/jcop.22741 PMC8916974

[cars70009-bib-0028] Coleman‐Jensen, A. , C. Gregory , and A. Singh . 2021. “A Household Food Security in the United States in 2019. USDA‐ERS Economic Research.” USDA. USDA‐ERS Report no. 275.

[cars70009-bib-0029] Donnelly, R. , B. A. Robinson , and D. Umberson . 2019. “Can Spouses Buffer the Impact of Discrimination on Depressive Symptoms? An Examination of Same‐Sex and Different‐Sex Marriages.” Society and Mental Health 9, no. 2: 192–210. 31223514 10.1177/2156869318800157 PMC6585990

[cars70009-bib-0030] Economic Research Service . 2012. “‘U.S. Adults Food Security Survey Module’ Three‐Stage Design, With Screeners.” Economic Research Service – USDA. https://www.ers.usda.gov/webdocs/DataFiles/50764/26623_ad2012.pdf?v=3672.4.

[cars70009-bib-0031] Foo, J. K. , and T. Doan . 2023. “The Impact of Sleep Quality on Mental Health in Working Australians: A Quasi‐Experimental Approach.” Social Science & Medicine 329: 116039. 37379637 10.1016/j.socscimed.2023.116039

[cars70009-bib-0032] Frazier, C. 2023. “Working Around the Clock: The Association Between Shift Work, Sleep Health, and Depressive Symptoms Among Midlife Adults.” Society and Mental Health 13, no. 2: 97–110. 37860107 10.1177/21568693231156452 PMC10586491

[cars70009-bib-0033] Freeman, D. , B. Sheaves , G. M. Goodwin , et al. 2017. “The Effects of Improving Sleep on Mental Health (OASIS): A Randomised Controlled Trial With Mediation Analysis.” Lancet Psychiatry 4, no. 10: 749–758. 28888927 10.1016/S2215-0366(17)30328-0 PMC5614772

[cars70009-bib-0034] Graham, C. , and G. Ciciurkaite . 2023. “The Risk for Food Insecurity and Suicide Ideation Among Young Adults in the United States: The Mediating Roles of Perceived Stress and Social Isolation.” Society and Mental Health 13, no. 1: 61–78.

[cars70009-bib-0035] Grandner, M. A. , N. J. Williams , K. L. Knutson , D. Roberts , and G. Jean‐Louis . 2016. “Sleep Disparity, Race/Ethnicity, and Socioeconomic Position.” Sleep Medicine 18: 7–18. 26431755 10.1016/j.sleep.2015.01.020 PMC4631795

[cars70009-bib-0036] Hale, L. , W. Troxel , and D. J. Buysse . 2020. “Sleep Health: An Opportunity for Public Health to Address Health Equity.” Annual Review of Public Health 41, no. 1: 81–99. 10.1146/annurev-publhealth-040119-094412 PMC7944938 31900098

[cars70009-bib-0037] Hayes, A. F. 2013. Introduction to Mediation, Moderation, and Conditional Process Analysis: A Regression‐Based Approach. 3rd ed. Guilford Publications.

[cars70009-bib-0038] Hisler, G. C. , and R. E. Brenner . 2019. “Does Sleep Partially Mediate the Effect of Everyday Discrimination on Future Mental and Physical Health?” Social Science & Medicine 221: 115–123. 30580073 10.1016/j.socscimed.2018.12.002

[cars70009-bib-0039] Jacob, L. , L. Smith , K. Kostev , et al. 2023. “Food Insecurity and Insomnia‐related Symptoms Among Adults From Low‐ and Middle‐income Countries.” Journal of Sleep Research 32, no. 4: e13852. 36808652 10.1111/jsr.13852

[cars70009-bib-0040] Johnson, D. , R. Thorpe , J. Mcgrath , W. Jackson , and C. Jackson . 2018. “Black–White Differences in Housing Type and Sleep Duration as Well as Sleep Difficulties in the United States.” International Journal of Environmental Research and Public Health 15, no. 4: 564. 29561769 10.3390/ijerph15040564 PMC5923606

[cars70009-bib-0041] Jordan, M. L. , R. Perez‐Escamilla , M. M. Desai , and T. Shamah‐Levy . 2016. “Household Food Insecurity and Sleep Patterns Among Mexican Adults: Results From ENSANUT‐2012.” Journal of Immigrant and Minority Health 18: 1093–1103. 26163336 10.1007/s10903-015-0246-5

[cars70009-bib-0042] Kiecolt‐Glaser, J. K. , and T. L. Newton . 2001. “Marriage and Health: His and Hers.” Psychological Bulletin 127, no. 4: 472–503. 11439708 10.1037/0033-2909.127.4.472

[cars70009-bib-0043] Lee, T.‐H. , J.‐H. Kuo , C.‐Y. Liu , et al. 2021. “Trajectory of Food Insecurity and Its Association With Longitudinal Mental Health and Sleep Outcomes in Adolescents From Economically Disadvantaged Families.” Nutrients 13, no. 5: 1696. 34067617 10.3390/nu13051696 PMC8157056

[cars70009-bib-0044] Mallinckrodt, B. , W. T. Abraham , M. Wei , and D. W. Russell . 2006. “Advances in Testing the Statistical Significance of Mediation Effects.” Journal of Counseling Psychology 53, no. 3: 372–378.

[cars70009-bib-0045] Masa, R. , S. Shangani , D. Baruah , and D. Operario . 2024. “The Association of Food Insecurity, Mental Health, and Healthcare Access and Use Among lesbian, Gay, and Bisexual Adults in the United States: Results From the 2021 National Health Interview Survey.” American Journal of Health Promotion 38, no. 1: 68–79. 37899588 10.1177/08901171231211134 PMC10748451

[cars70009-bib-0046] Mazloomi, S. N. , S. Talebi , M. Kazemi , et al. 2023. “Food Insecurity Is Associated With the Sleep Quality and Quantity in Adults: A Systematic Review and Meta‐Analysis.” Public Health Nutrition 26, no. 4: 792–802. 36416108 10.1017/S1368980022002488 PMC10131157

[cars70009-bib-0047] Meijs, L. , F. Handy , F. J. Simons , and L. Roza . 2020. “A Social Innovation: Addressing Relative Food Insecurity and Social Exclusion.” VOLUNTAS: International Journal of Voluntary and Nonprofit Organizations 31: 894–906.

[cars70009-bib-0048] Nagata, J. M. , K. Palar , H. C. Gooding , et al. 2019. “Food Insecurity Is Associated With Poorer Mental Health and Sleep Outcomes in Young Adults.” Journal of Adolescent Health 65, no. 6: 805–811. 10.1016/j.jadohealth.2019.08.010 PMC6874757 31587956

[cars70009-bib-0049] National Center for Health Statistics (NCHS) . 2023. “National Health Interview Survey, 2022 Survey Description.” National Center for Health Statistics. https://ftp.cdc.gov/pub/Health_Statistics/NCHS/Dataset_Documentation/NHIS/2022/srvydesc‐508.pdf.

[cars70009-bib-0050] Osei Bonsu, E. , M. Afetor , L. Munkaila , et al. 2023. “Association of Food Insecurity and Sleep Difficulty Among 189,619 School‐Going Adolescents: A Study From the Global In‐School Students Survey.” Frontiers in Public Health 11: 1212254. 37501946 10.3389/fpubh.2023.1212254 PMC10369053

[cars70009-bib-0051] Pandi‐Perumal, S. R. , J. M. Monti , D. Burman , et al. 2020. “Clarifying the Role of Sleep in Depression: A Narrative Review.” Psychiatry Research 291: 113239. 32593854 10.1016/j.psychres.2020.113239

[cars70009-bib-0052] Patel, P. , K. A. Patte , K. Storey , S. T. Leatherdale , and R. Pabayo . 2024. “Exploring the Association Between Income Inequality and Sleep in Canadian Adolescents: a Path Analysis Approach.” Sleep Health 10, no. 4: 410–417. 38714386 10.1016/j.sleh.2024.03.008

[cars70009-bib-0053] Pearlin, L. I. , and A. Bierman . 2013. “Current Issues and Future Directions in Research Into the Stress Process.” In Handbook of the Sociology of Mental Health, edited by C. S. Aneshensel , J. C. Phelan , and A. Bierman , 325–340. Springer.

[cars70009-bib-0054] Pineau, C. , P. L. Williams , J. Brady , M. Waddington , and L. Frank . 2021. “Exploring Experiences of Food Insecurity, Stigma, Social Exclusion, and Shame Among Women in High‐Income Countries: A Narrative Review.” Canadian Food Studies/La Revue Canadienne Des études Sur L'alimentation 8, no. 3: 107–124.

[cars70009-bib-0055] Poole‐Di Salvo, E. , E. J. Silver , and R. E. Stein . 2016. “Household Food Insecurity and Mental Health Problems Among Adolescents: What Do Parents Report?” Academic Pediatrics 16, no. 1: 90–96. 26530851 10.1016/j.acap.2015.08.005

[cars70009-bib-0056] Purdam, K. , E. A. Garratt , and A. Esmail . 2016. “Hungry? Food Insecurity, Social Stigma and Embarrassment in the UK.” Sociology 50, no. 6: 1072–1088.

[cars70009-bib-0057] Reczek, C. , M. B. Thomeer , L. Gebhardt‐Kram , and D. Umberson . 2020. “‘Go See Somebody’: How Spouses Promote Mental Health Care.” Society and Mental Health 10, no. 1: 80–96. 33224557 10.1177/2156869319834335 PMC7676732

[cars70009-bib-0058] Rosenström, T. , M. Jokela , S. Puttonen , et al. 2012. “Pairwise Measures of Causal Direction in the Epidemiology of Sleep Problems and Depression.” PLoS ONE 7, no. 11: e50841. 23226400 10.1371/journal.pone.0050841 PMC3511346

[cars70009-bib-0059] Ross, C. E. 1991. “Marriage and the Sense of Control.” Journal of Marriage and the Family 53, no. 4: 831–838.

[cars70009-bib-0060] Saunders, R. , Y. Liu , H. Delamain , et al. 2023. “Examining Bi‐Directional Change in Sleep and Depression Symptoms in Individuals Receiving Routine Psychological Treatment.” Journal of Psychiatric Research 163: 1–8. 37178582 10.1016/j.jpsychires.2023.05.007 PMC10643991

[cars70009-bib-0061] Scott, A. J. , T. L. Webb , M. Martyn‐St James , G. Rowse , and S. Weich . 2021. “Improving Sleep Quality Leads to Better Mental Health: A Meta‐Analysis of Randomised Controlled Trials.” Sleep Medicine Reviews 60: 101556. 34607184 10.1016/j.smrv.2021.101556 PMC8651630

[cars70009-bib-0062] Shepherd, D. L. 2022. “Food Insecurity, Depressive Symptoms, and the Salience of Gendered Family Roles During the COVID‐19 Pandemic in South Africa.” Social Science & Medicine 301: 114830. 35367907 10.1016/j.socscimed.2022.114830 PMC8882481

[cars70009-bib-0063] Simon, R. W. 2002. “Revisiting the Relationships Among Gender, Marital Status, and Mental Health.” American Journal of sociology 107, no. 4: 1065–1096. 10.1086/339225 12227382

[cars70009-bib-0064] Smith, J. , H. Stevens , A. A. Lake , S. Teasdale , and E. L. Giles . 2023. “Food Insecurity in Adults With Severe Mental Illness: A Systematic Review With Meta‐Analysis.” Journal of Psychiatric and Mental Health Nursing 31, no. 2: 133–151. 37621069 10.1111/jpm.12969

[cars70009-bib-0065] Still, D. 2021. “Romantic Relationship Quality and Suicidal Ideation in Young Adulthood.” Society and Mental Health 11, no. 2: 134–148.

[cars70009-bib-0066] Thoits, P. A. , and B. G. Link . 2016. “Stigma Resistance and Well‐Being Among People in Treatment for Psychosis.” Society and Mental Health 6, no. 1: 1–20.

[cars70009-bib-0067] Thomas, M. , D. P. Miller , and T. W. Morrissey . 2019. “Food Insecurity and Child Health.” Pediatrics 144, no. 4: e20190397. 31501236 10.1542/peds.2019-0397

[cars70009-bib-0068] Troxel, W. M. , A. Haas , B. Ghosh‐Dastidar , et al. 2020. “Food Insecurity Is Associated With Objectively Measured Sleep Problems.” Behavioral Sleep Medicine 18, no. 6: 719–729. 31545653 10.1080/15402002.2019.1669605 PMC8152928

[cars70009-bib-0069] Umberson, D. 1992. “Gender, Marital Status and the Social Control of Health Behavior.” Social Science & Medicine 34, no. 8: 907–917. 1604380 10.1016/0277-9536(92)90259-s

[cars70009-bib-0070] Walker, E. R. , J. R. Cummings , J. M. Hockenberry , and B. G. Druss . 2015. “Insurance Status, Use of Mental Health Services, and Unmet Need for Mental Health Care in the United States.” Psychiatric Services 66, no. 6: 578–584. 25726980 10.1176/appi.ps.201400248 PMC4461054

[cars70009-bib-0071] Wheaton, B. , and S. Montazer . 2010. “Stressors, Stress, and Distress.” In A Handbook for the Study of Mental Health: Social Contexts, Theories, and Systems, edited by T. L. Scheid and E. R. Wright , 171–199. Cambridge University Press.

[cars70009-bib-0072] Wight, V. , N. Kaushal , J. Waldfogel , and I. Garfinkel . 2014. “Understanding the Link Between Poverty and Food Insecurity Among Children: Does the Definition of Poverty Matter?” Journal of Children and Poverty 20, no. 1: 1–20. 25045244 10.1080/10796126.2014.891973 PMC4096937

[cars70009-bib-0073] Williams, K. 2003. “Has the Future of Marriage Arrived? A Contemporary Examination of Gender, Marriage, and Psychological Well‐Being.” Journal of Health and Social Behavior 44, no. 4: 470–487. 15038144 PMC4018193

[cars70009-bib-0074] Williams, M. R. , and D. P. Do . 2021. “The Compounded Burden of Poverty on Mental Health for People With Disabilities.” Social Work in Public Health 36, no. 4: 419–431. 33832403 10.1080/19371918.2021.1905579

